# Everyday decision-making in later life: the role of cognitive reserve and cognitive functions

**DOI:** 10.3389/fpsyg.2026.1841011

**Published:** 2026-06-11

**Authors:** Katharina Thaler, Johanna Büchel, Elisabeth Goettfried, Marie-Theres Pertl, Margarete Delazer, Iris Unterberger, Atbin Djamshidian, Laura Zamarian

**Affiliations:** 1 Department of Neurology, Medical University Innsbruck, Innsbruck, Austria; 2 Private University in the Principality of Liechtenstein (UFL), Triesen, Liechtenstein; 3 Department of Neuropsychology, Rehabilitation Clinic Walzenhausen, Clinic Group Valens, Walzenhausen, Switzerland

**Keywords:** cognitive functions, cognitive reserve, conflicting information, decision-making, frequencies, health numeracy, moderation, percentages

## Abstract

**Objectives:**

This study aimed to investigate decision-making competence of midlife and older adults, and its relationship with cognitive reserve and cognitive functions.

**Methods:**

A total of 120 healthy adults (aged 50 to 90 years) completed a decision-making task and various neurocognitive tests. In the decision-making task, participants encountered everyday scenarios with two options, including relevant and irrelevant numerical information, formatted as frequencies or percentages. In congruent trials, the relevant and irrelevant information pointed to the same decision, while in incongruent trials they pointed to opposing decisions. Cognitive reserve (CR) was measured using a composite index reflecting lifetime education, professional experiences, and engagement in cognitively demanding leisure activities.

**Results:**

Participants demonstrated higher accuracy in congruent trials than in incongruent ones, with this difference being more pronounced among individuals with lower CR. Additionally, decision-making performance decreased with increasing age for both number formats, with a steeper decline observed in trials involving frequencies. CR, along with measures of judgement, mental calculation, and health numeracy, accounted for 41% of the variance in decision-making performance. CR also moderated the relationship between decision-making performance and health numeracy: participants with poorer numeracy skills performed accurately if their CR level was high.

**Discussion:**

This study confirms the positive relationship between CR and decision-making ability in advanced age. Higher levels of CR are linked to reduced decline in health numeracy with age and lower susceptibility to conflicting information during decision-making.

## 1 Introduction

In modern society, individuals regularly encounter numerical information in various contexts, from inflation percentages and global temperature increases to vaccine efficacy rates. Strong numerical skills are essential for accurate understanding and use of this data. Longitudinal studies show that some numerical skills tend to decline in later middle age and deteriorate further with advancing age (Best et al., 2022). Older adults are slower than younger adults in solving simple and complex calculations (Avcil and Artemenko, 2025; Zamarian et al., 2007), although their accuracy remains comparable (Avcil and Artemenko, 2025; Goettfried et al., 2025). Challenges also arise in employing computation strategies, with older individuals showing less efficiency and flexibility (Hinault and Lemaire, 2020). Additionally, older adults often struggle more than young adults with ratio concepts (Hinault and Lemaire, 2020; Delazer et al., 2013). Cognitive processes such as working memory and interference inhibition are crucial for number processing (Hohol et al., 2017), suggesting that declines in numeracy may stem from age-related deterioration in these domain-general cognitive abilities. Supporting this, Delazer et al. (2013) [see also Zamarian et al. (2020)] found that the relationship between age and numeracy is partially mediated by executive functions.

In healthcare, information is often conveyed not only through basic quantitative data but also through complex concepts like probabilities, proportions, and survival rates, which require higher skill levels. Health numeracy refers to an individual’s “capacity to access, process, interpret, communicate, and act on numerical, quantitative, graphical, biostatical, and probabilistic health information needed to make effective health decisions” (Golbeck et al., 2005). Research shows that many people lack the numerical skills required to evaluate risks and make informed decisions (Garcia-Retamero et al., 2019). Even highly educated individuals (Lipkus et al., 2001) and healthcare professionals (Gigerenzer et al., 2007) often struggle with probability questions and converting frequencies to percentages or vice-versa. The format of numerical information appears to play a critical role. Pertl et al. (2017) found that older individuals perform better when information is presented as percentages rather than frequencies in a health decision-making task. Similarly, Woloshin and Schwartz (2011) reported that percentages lead to more correct responses than frequencies, regardless of education or numeracy levels. However, results are mixed; some studies demonstrate that individuals perform better with frequencies (Galesic et al., 2009), others find no difference (for a discussion, see Reyna and Brainerd (2008); Reyna et al. (2009)).

Studies show that low numeracy is associated with inaccurate perceptions of health-related risks and benefits, suboptimal medical decision-making, and poor health outcomes (Reyna et al., 2009). For instance, low numerate individuals often struggle to understand the risks associated with treatments (Gardner et al., 2011). They also tend to have less positive attitudes toward screening procedures (Smith et al., 2016). Low numeracy affects self-management of chronic conditions such as diabetes (Cavanaugh et al., 2008) and increases susceptibility to framing effects, i.e., to whether information is presented in positive or negative terms (Zamarian et al., 2010). Low numerate individuals are also more likely to delay seeking medical care, significantly increasing their risk of severe disability or death (Reyna et al., 2009). Low numeracy levels have also been linked to poorer financial decision-making. For instance, a recent study (Danesin et al., 2022) showed that lower financial decision-making ability is associated with poorer performance in mental calculations involving percentages in real-life contexts among healthy older adults. A further study (Bangma et al., 2017) revealed that numeracy is the best predictor of financial decision-making ability throughout adulthood.

These aspects become particularly relevant in advanced age, as individuals increasingly have to make health and financial decisions for themselves or their spouses. Research on aging and decision-making shows mixed results; some studies find age-related changes, while others do not (Strough and de Bruine Bruin, 2020). It appears that age-related differences in decision-making depend on the decision type and task demands. Older adults perform similarly to younger adults on decision-making tasks under risk conditions (Zamarian et al., 2008), on tasks involving resistance to sunk costs (Del Missier et al., 2020), or on tasks requiring judgement and recognition of social norms (de Bruine Bruin et al., 2012). Conversely, they are more susceptible to framing effects (Zamarian et al., 2010; de Bruine Bruin et al., 2012), may use decision rules inconsistently (Rosi et al., 2019), and tend to perform more poorly in decision-making tasks under ambiguity (Zamarian et al., 2008). Research suggests that these differences may be linked to age-related declines in cognitive abilities such as working memory, processing speed, and executive functions (Strough and de Bruine Bruin, 2020; Henninger et al., 2010; Schiebener and Brand, 2015). However, decades of experience and accumulated knowledge may compensate for some cognitive declines, enabling older adults to make informed decisions and providing advantages in certain situations (Li et al., 2013).

In this context, cognitive reserve (CR) is suggested as a key factor in maintaining cognitive functioning in older age. CR is a theoretical construct defined as “the adaptability […] of cognitive processes that helps to explain differential susceptibility of cognitive abilities or day-to-day function to brain aging, pathology or insult” (Stern et al., 2020). It results from a combination of genetic factors and lifetime experiences, including education, profession, leisure activities, and social engagement (Stern et al., 2020). Higher CR has been linked to better preservation of various cognitive abilities, including executive functions, important for numerical processing and decision-making (Zamarian et al., 2020; Khalaila et al., 2024; Oosterman et al., 2021). Research indicates that education, serving as a proxy for CR, correlates with improved numerical skills in older adults, with those having higher educational levels outperforming those with less education on complex numerical tasks (Delazer et al., 2013; Zamarian et al., 2020; Arcara et al., 2017). Regarding decision-making, evidence remains limited. Wilson et al. (2023) found that higher purpose in life was associated with a slower decline in deliberative/analytic decision-making, while greater lifetime cognitive activity was linked to a slower increase in suboptimal decision-making preferences with age.

To enhance our understanding of decision-making in midlife and older adults, this study investigated the competence of adults aged fifty and older in everyday decision-making scenarios involving complex numerical information. We also examined how cognitive functions and CR relate to decision-making. Specifically, we assessed whether higher levels of CR—measured through years of formal education and a composite index including lifetime curricular and extracurricular education, occupational experiences, and engagement in cognitively demanding leisure activities—are associated with reduced age-related changes in decision-making. The decision-making task employed included both relevant and irrelevant numerical information, presented in two formats: frequencies and percentages. This experimental design allowed us to investigate: 1) whether midlife and older adults experience fewer difficulties in identifying the more advantageous option when the relevant and irrelevant numerical information are congruent (congruent trials: the two numerical information align, pointing to the same decision; incongruent trials: the two numerical information do not align, pointing to opposing decisions; for examples, see Supplementary Materials); and 2) whether they are influenced by the number format. Additionally, this study aimed to assess 3) the associations between decision-making competence and demographic variables such as age, gender, and CR; 4) the relationships with neurocognitive variables, including episodic memory, attention, executive functions, and both basic and complex numerical skills; 5) potential predictors of decision-making competence; and 6) the moderating effect of CR on the relationship between decision-making competence and neurocognitive variables. Our focus was on cognitive functions that tend to decline with aging and are relevant for performance in decision-making tasks involving complex numerical information.

We hypothesised that 1) midlife and older adults demonstrate greater accuracy in identifying the more advantageous option when it is presented through congruent numerical information. Given the mixed results in the existing literature, it remains to be investigated 2) whether midlife and older adults differ in their performance when processing frequencies versus percentages. Additionally, we expected that 3) higher decision-making accuracy correlates with younger age and greater CR. The potential association between decision-making performance and gender remains an open question, as previous research yielded inconsistent findings. While some studies reported differences between men and women (Blais and Weber, 2001; Villanueva-Moya and Expósito, 2021), others did not (Schiebener and Brand, 2015). Considering the complexity of the numerical information involved, we also assumed that decision-making performance is 4) associated with, and 5) predicted by, executive functions and numerical competence, besides CR. Finally, we hypothesised that 6) CR moderates the relationships between decision-making competence and cognitive functioning, with higher CR being associated with better decision-making outcomes, even when individuals exhibit poorer executive functions and numerical skills.

## 2 Materials and methods

### 2.1 Participants

In total, 120 healthy independently living adults participated in this study. Inclusion criteria required participants to be 50 years of age or older, to have a Mini-Mental State Examination (MMSE) score of 27 or higher, and to demonstrate good reading comprehension skills as indicated by scores on a screening test. Exclusion criteria were a history of severe neurological or psychiatric disorders, substance abuse, the intake of medications that can affect cognition, and major surgeries under general anaesthesia within the past 2 years. None of the participants were excluded based on these criteria.

### 2.2 Standard protocol approvals and informed consent

This study is part of a larger project on numeracy and decision-making across adulthood. It was approved by the ethics committee of the Medical University of Innsbruck with the approval number 1187/2020, dated 13.07.2020, and conforms to the World Medical Association Declaration of Helsinki for studies involving human subjects. Written informed consent was obtained from all individuals before participation.

### 2.3 Measures

#### 2.3.1 Cognitive reserve

As measures of CR, we considered both years of formal education and scores from the Cognitive Reserve Index Questionnaire [CRIq, paper-and-pencil German version; Nucci et al. (2012)]. This questionnaire collects information about an individual’s entire life in relation to education, profession, and leisure time. The interviewer assigns points based on years of education (including both curricular and extracurricular courses), the level and duration of occupational activities, and the duration of frequently practiced cognitively demanding leisure activities (e.g., reading newspapers, driving a car, or going to the cinema). An overall score is calculated to classify an individual’s cognitive reserve index (CRI). The CRI can be categorised as low (<70 points), medium-low (70–84 points), medium (85–114 points), medium-high (115–130 points), and high (>130 points).

#### 2.3.2 Neuropsychological background tests and numeracy tasks

All participants underwent a comprehensive neuropsychological battery including validated standardised cognitive tests assessing episodic memory, verbal attention span, and executive functions. Additionally, participants completed a cognitive reflection test, a framing task, a mental calculation task, and a health numeracy task (for a detailed description, see Supplementary Materials).

#### 2.3.3 Decision-making task

The Everyday Decision-Making Task (hereafter, EDDM) was developed by our working group as a modified version of the decision-making task by Pertl et al. (2017). The EDDM task consists of 12 short text problems focusing on practical health-related decisions (for examples, see Supplementary Materials). For each decision, participants are presented with two options (alternative A and alternative B) and are asked to choose the more advantageous one. Each alternative contains two pieces of numerical information: one that is relevant to the decision (e.g., “Medication A improves symptoms in 95% of cases”) and another that is irrelevant (e.g., “80% of the patients find the name of medication A easy to remember”). The relevant numerical information is presented either as percentages (e.g., 95%) or as frequencies (e.g., 95 out of 100), while the irrelevant information is consistently presented as percentages. There are a total of six congruent trials and six incongruent trials. In the congruent trials, the numerical values for both relevant and irrelevant information associated with the advantageous option are greater than those for the disadvantageous option. Thus, both numerical values align and point to the same decision. Conversely, in the incongruent trials, the numerical value for the relevant information is greater for the advantageous option, while the numerical value for the irrelevant information is greater for the disadvantageous option. Therefore, the relevant and irrelevant numerical information do not align and point to diverging options. To select the more favourable option, participants need to correctly identify which information is relevant to the decision. This manipulation makes the incongruent condition inherently more difficult than the congruent condition. In both conditions, a correct response is defined as selecting the option associated with the greater relevant information. In addition to choosing the more advantageous option, participants are required to indicate on a coloured 5-point scale ranging from yellow (“not at all”) to dark green (“completely”) how much they preferred the chosen alternative over the other. For analysis purposes, answers are recoded into values ranging from 1 (yellow) to 5 (dark green). To enhance engagement and concentration, participants are given 45 s. to make their choice and indicate their preference. In this study, response times were not collected and analyses focused on the number of correctly identified advantageous options (hereafter referred to as the “accuracy score”). Regarding preference ratings, we report the median score.

### 2.4 Statistical analysis

Statistical analyses were carried out with IBM SPSS Version 29.0 (SPSS, Chicago, IL; RRID: SCR_002865), using parametric statistical methods. We first conducted a Pearson correlation analysis to examine the relationship between demographic variables and accuracy scores in the EDDM task. The significant demographic variables identified from this analysis were included as covariates in a repeated-measures Analysis of Covariance (ANCOVA). This ANCOVA examined accuracy scores in the EDDM task, with congruency (congruent vs. incongruent) and number format (frequencies vs. percentages) as within-subjects factors. Additional Pearson correlation analyses were performed to investigate the relationships between accuracy scores in the EDDM task, preference rates, and neurocognitive variables. In all correlation analyses, we applied a false discovery rate (FDR) correction to address the issue of multiple testing. A hierarchical regression model was conducted to identify potential predictors of accuracy scores in the EDDM task, with the neurocognitive variables included in the first step and the demographic variables in the second step. This analysis was restricted to a maximum of 14 predictors to ensure sufficient statistical power to detect effect sizes (f2) between 0.10 and 0.15 with a sample size of 120 participants (two-sided, *α* = 0.05, power = 0.95; G*Power version 3.1.9.7 (Faul et al., 2007); RRID: SCR_013726). Potential neurocognitive predictors and demographic variables were selected based on the results of the correlation analyses and the ANCOVA. Subsequently, we conducted moderation analyses to investigate the influence of neurocognitive measures and CR, along with their potential interactive effects on decision-making performance. This analysis was performed separately for each neurocognitive measure that emerged as a significant predictor in the hierarchical regression analysis. In each moderation analysis, CRI was entered as moderator. The moderation analyses were conducted on raw scores via the PROCESS macro for SPSS (version 4.2 (Hayes, 2022); RRID: SCR_021369), which automatically transforms the predictors using grand mean centering. The Johnson-Neyman technique was applied to determine the regions of significance in case of significant interactive effects. Overall, significance level was set at *α* = 0.05.

## 3 Results

### 3.1 Sample characteristics and neurocognitive outcomes

The mean age was 63.0 years (SD = 9.9, range 50–90), and the mean duration of education was 12.3 years (SD = 3.2, range 8–20). Sixty-two individuals were female (51.7%). The mean CRI score was 121.9 (SD = 14.4, range 84–160). Specifically, no participants fell in the low CRI category; one participant (0.8%) was in the medium-low category, 36 participants (30%) in the medium category, 50 participants (41.7%) in the medium-high category, and 33 participants (27.5%) in the high category. Performance on neuropsychological tests and questionnaires are presented in Supplementary Table S1. In all cognitive tests, group scores were within the average range of standardised norms. Group scores on the questionnaire assessing anxiety and depression symptoms were also within the normal range.

### 3.2 General performance on the EDDM task

Detailed scores are reported in Supplementary Table S2. In the EDDM task, 55 people (45.8%) achieved the maximum score of 12 points, and none scored zero points. The overall accuracy was high, with a mean of 10.9 (SD = 1.5, range 5–12). Preference ratings averaged 4.4 (SD = 0.7), on a 1–5 scale. A significant positive correlation was observed between accuracy scores and preference ratings (*r* = 0.22, *p* = 0.015), indicating a moderate alignment between the individuals’ objective decision-making performance and their subjective ratings.

### 3.3 Relationships between decision-making performance and demographic variables

We found a significant negative correlation between decision-making performance and age (*r* = −0.36, *p* < 0.001), as well as significant positive correlations between decision-making performance and CR measures (education: *r* = 0.21, *p* = 0.023; CRI: *r* = 0.31, *p* < 0.001). Higher accuracy scores in the EDDM task were associated with younger age, higher formal education, and a higher CRI. The correlation between gender (male = 0, female = 1) and accuracy was negative but failed to be statistically significant (*r* = −0.18, *p* = 0.056, 95% CI −0.34 to 0.01).

### 3.4 Relationships between decision-making performance and neurocognitive variables

Results are shown in Supplementary Figure F1. Overall, we found significant correlations (all *p* < 0.05) with all but one measure, namely phonemic verbal fluency. Higher performance on the EDDM task was associated with better episodic memory, verbal attention span, executive functions, cognitive reflection, and basic and more complex numerical skills, as well as with reduced framing effects.

### 3.5 Effects of congruency, number format, and demographic variables on decision-making performance

**Figure 1:**
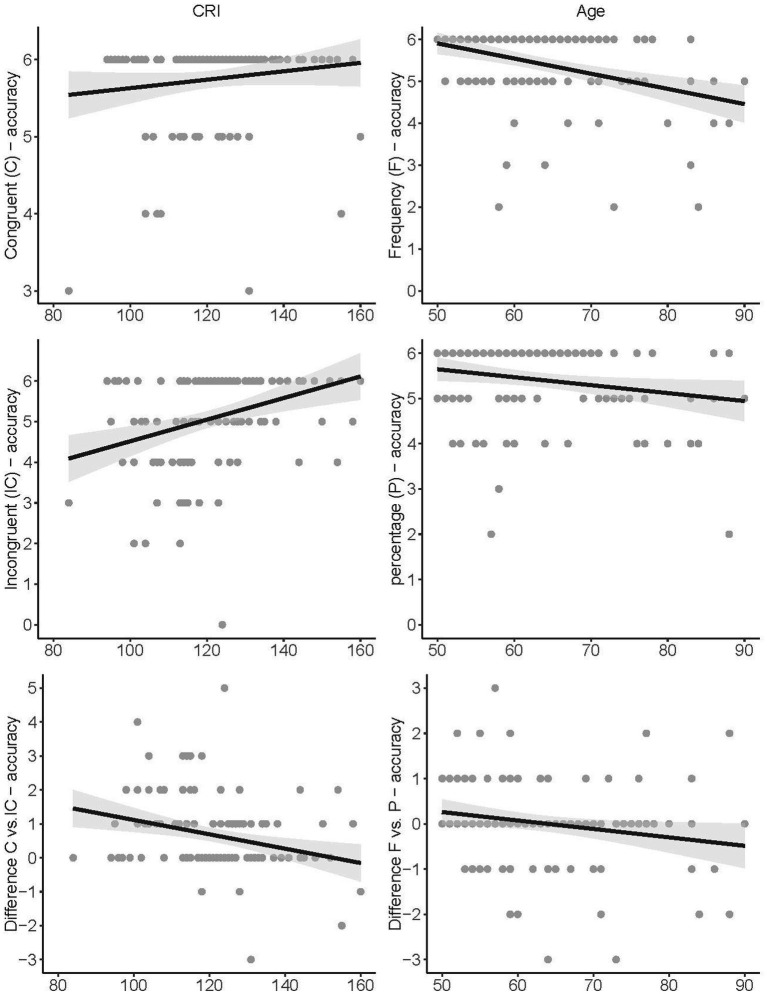
Correlation plots between measures of performance on the EDDM and demographic variables [CRI, left panel **(A)**; age, right panel **(B)**]. In each plot, the black line shows the linear function that best fits the data, while the shadowed area indicates the confidence interval set at 95%. EDDM, Everyday decision-making task; CRI, Cognitive reserve index; C, congruent condition; IC, incongruent condition; F, frequency trials; P, percentage trials.

A repeated-measures ANCOVA was conducted to examine the effects of congruency and number format on accuracy scores while controlling for age, education, and CRI. The results revealed a significant main effect of congruency [*F*(1,116) = 9.30, *p* = 0.003, ηp2 = 0.07], with participants demonstrating greater accuracy in congruent trials than in incongruent trials. In contrast, there was no significant main effect of number format [*F*(1,116) = 3.53, *p* = 0.063, ηp2 = 0.03]. Two covariates showed significant effects: accuracy scores on the EDDM task were significantly influenced by age [*F*(1,116) = 20.17, *p* < 0.001, ηp2 = 0.15] and CRI [*F*(1,116) = 11.43, *p* < 0.001, ηp2 = 0.09]. The covariate education was not significant [*F*(1,116) = 0.23, *p* = 0.635, ηp2 = 0.00]. There was a significant interaction between congruency and CRI [*F*(1,116) = 5.25, *p* = 0.024, ηp2 = 0.04], but not between congruency and number format [*F*(1,116) = 0.35, *p* = 0.556, ηp2 = 0.00], congruency and age [*F*(1,116) = 0.01, *p* = 0.945, ηp2 = 0.00], or congruency and education [*F*(1,116) = 0.06, *p* = 0.807, ηp2 = 0.00]. The interaction between number format and CRI [*F*(1,116) = 0.85, *p* = 0.359, ηp2 = 0.01] and between number format and education [*F*(1,116) = 0.41, *p* = 0.526, ηp2 = 0.00] did not reach significance, while the interaction between number format and age was marginally non-significant [*F*(1,116) = 3.90, *p* = 0.051, ηp2 = 0.03]. None of the three-way interactions were significant: congruency x number format x age [*F*(1,116) = 1.00, *p* = 0.319, ηp2 = 0.01]; congruency x number format x CRI [*F*(1,116) = 0.08, *p* = 0.773, ηp2 = 0.00]; and congruency x number format x education [*F*(1,116) = 0.07, *p* = 0.790, ηp2 = 0.00]. A detailed analysis of the significant interactions showed that a larger congruency effect (where accuracy scores in congruent trials were higher than those in incongruent trials) was associated with a lower CRI (*r* = −0.28, *p* = 0.002), and that a larger number format effect (where accuracy scores with frequencies were higher than those with percentages) was associated with a younger age (*r* = −0.20, *p* = 0.033). A closer examination of these effects revealed a significant positive correlation of CRI with accuracy scores in the incongruent condition (*r* = 0.33, *p* < 0.001), but not with accuracy scores in the congruent condition [*r* = 0.13, *p* = 0.151; Figure 1, left panel (A)]. Furthermore, age was significantly and negatively correlated with accuracy scores for both number formats, although the decline in performance with age was steeper in the case of frequencies (*r* = −0.39, *p* < 0.001) compared to percentages [*r* = −0.20, *p* = 0.027; Figure 1, right panel (B)].

### 3.6 Cognitive and demographic predictors of decision-making performance

The results of the hierarchical regression analysis are summarised in Table 1. Model 1 was significant [*F*(12,106) = 6.52, *p* < 0.001] and explained 36.0% of variance, with measures of judgement, mental calculation, and health numeracy emerging as significant predictors of performance on the EDDM task. Model 2, where demographic variables were added to the predictors of Model 1, resulted in a significant increase in explained variance [Δ*R^2^* = 0.052, *F*(2, 104) = 5.22, *p* = 0.007]. This model accounted for 40.7% of the variance and was significant [*F*(14, 104) = 6.78, *p* < 0.001], with measures of judgement, mental calculation, health numeracy, and CRI being the significant predictors. The Variance Inflation Factor (VIF) indicated no significant multicollinearity between the predictors.

**Table 1:** Coefficients of a hierarchical regression analysis where performance on the EDDM (accuracy score) is the dependent variable (*N* = 120).

Model		Unstandardized coefficients		Standardized coefficients	*t*	*p*	Collinearity		95% CI	
		B	SE	Beta (*β*)			B	SE	lower	upper
1	(Intercept)	−3.065	2.482		−1.235	0.220			−7.986	1.856
	Verbal free recall	0.009	0.089	0.010	0.097	0.923	0.543	1.842	−0.167	0.185
	Verbal attention span	−0.016	0.064	−0.024	−0.253	0.801	0.583	1.716	−0.144	0.112
	Verbal working memory	−0.020	0.073	−0.028	−0.278	0.782	0.528	1.893	−0.165	0.124
	Psychomotor speed	0.000	0.011	0.005	0.046	0.963	0.526	1.902	−0.021	0.022
	Cognitive flexibility	−0.001	0.005	−0.023	−0.174	0.862	0.314	3.182	−0.011	0.009
	Planning	0.051	0.067	0.068	0.767	0.445	0.701	1.427	−0.081	0.183
	Interference inhibition	−0.007	0.016	−0.048	−0.454	0.651	0.493	2.027	−0.038	0.024
	Judgement	0.217	0.075	0.228	2.906	**0.004**	0.879	1.138	0.069	0.366
	Framing	−0.014	0.099	−0.012	−0.144	0.886	0.725	1.379	−0.21	0.182
	Cognitive reflection	−0.025	0.127	−0.018	−0.197	0.844	0.652	1.534	−0.277	0.227
	Mental calculation	0.493	0.143	0.298	3.439	**<0.001**	0.721	1.387	0.209	0.777
	Health numeracy	0.300	0.098	0.332	3.077	**0.003**	0.465	2.149	0.107	0.494
2	(Intercept)	−5.363	2.766		−1.939	0.055			−10.848	0.123
	Verbal free recall	−0.042	0.089	−0.047	−0.471	0.639	0.502	1.992	−0.218	0.134
	Verbal attention span	0.011	0.063	0.017	0.182	0.856	0.565	1.771	−0.114	0.137
	Verbal working memory	−0.012	0.071	−0.017	−0.173	0.863	0.522	1.918	−0.152	0.128
	Psychomotor speed	0.004	0.011	0.038	0.367	0.715	0.471	2.125	−0.017	0.025
	Cognitive flexibility	0.000	0.005	0.002	0.018	0.986	0.307	3.258	−0.01	0.01
	Planning	0.080	0.065	0.105	1.228	0.222	0.686	1.457	−0.049	0.208
	Interference inhibition	−0.008	0.016	−0.052	−0.497	0.620	0.458	2.181	−0.039	0.023
	Judgement	0.221	0.077	0.232	2.879	**0.005**	0.772	1.295	0.069	0.373
	Framing	−0.002	0.095	−0.001	−0.018	0.986	0.722	1.385	−0.191	0.187
	Cognitive reflection	−0.082	0.124	−0.059	−0.662	0.510	0.636	1.573	−0.328	0.164
	Mental calculation	0.535	0.140	0.324	3.828	**<0.001**	0.704	1.421	0.258	0.812
	Health numeracy	0.213	0.098	0.235	2.176	**0.032**	0.429	2.328	0.019	0.407
	Age	−0.018	0.017	−0.122	−1.091	0.278	0.402	2.487	−0.051	0.015
	CRI	0.026	0.008	0.256	3.168	**0.002**	0.771	1.298	0.01	0.042

EDDM, Everyday decision-making task; CRI, Cognitive reserve index; CI, confidence interval. Significant results are highlighted in bold.

### 3.7 Interactive effects of cognitive reserve and cognitive functions on decision-making performance

Results are reported in Table 2. The first two moderation analyses tested whether CRI moderates the relationships between (1) judgement and decision-making performance, and (2) mental calculation and decision-making performance. The first model was significant [*R^2^* = 0.228, *F*(3, 116) = 8.95, *p* < 0.001], with significant main effects of judgement and CRI but no significant interaction. The second model was also significant [*R^2^* = 0.348, *F*(3, 116) = 14.78, *p* < 0.001], showing significant main effects of mental calculation and CRI but no significant interaction. Thus, CRI did not moderate either relationship.

**Table 2:** Moderation analyses with a specific cognitive variable (A: judgement, B: mental calculation, C: health numeracy) as independent variable, CRI as moderator, and accuracy scores in the EDDM as dependent variable.

	Model	B	SE B	*t*	*p*	95% CI [lower; upper]
A	Constant	10.85	0.12	87.67	<0.001	[10.60, 11.09]
	Judgement (centered)	0.36	0.11	3.13	0.002	[0.13, 0.58]
	CRI (centered)	0.04	0.02	2.60	0.01	[0.01, 0.06]
	Judgement x CRI	0.00	0.01	0.01	0.991	[−0.01, 0.01]
B	Constant	10.87	0.12	91.60	<0.001	[10.64, 11.11]
	Mental calculation (centered)	0.75	0.16	4.78	<0.001	[0.44, 1.06]
	CRI (centered)	0.02	0.01	2.69	0.008	[0.01, 0.04]
	Mental calculation x CRI	−0.01	0.01	−1.54	0.125	[−0.03, 0.00]
C	Constant	10.92	0.13	86.72	<0.001	[10.68, 11.17]
	Health numeracy (centered)	0.40	0.06	6.33	<0.001	[0.27, 0.52]
	CRI (centered)	0.02	0.01	1.78	0.08	[−0.00, 0.03]
	Health numeracy x CRI	−0.01	0.00	−1.99	0.049	[−0.02, 0.00]

EDDM, Everyday decision-making task; CRI, Cognitive reserve index; CI, confidence interval. Unstandardized regression coefficients for the variables included in the model.

**Figure 2:**
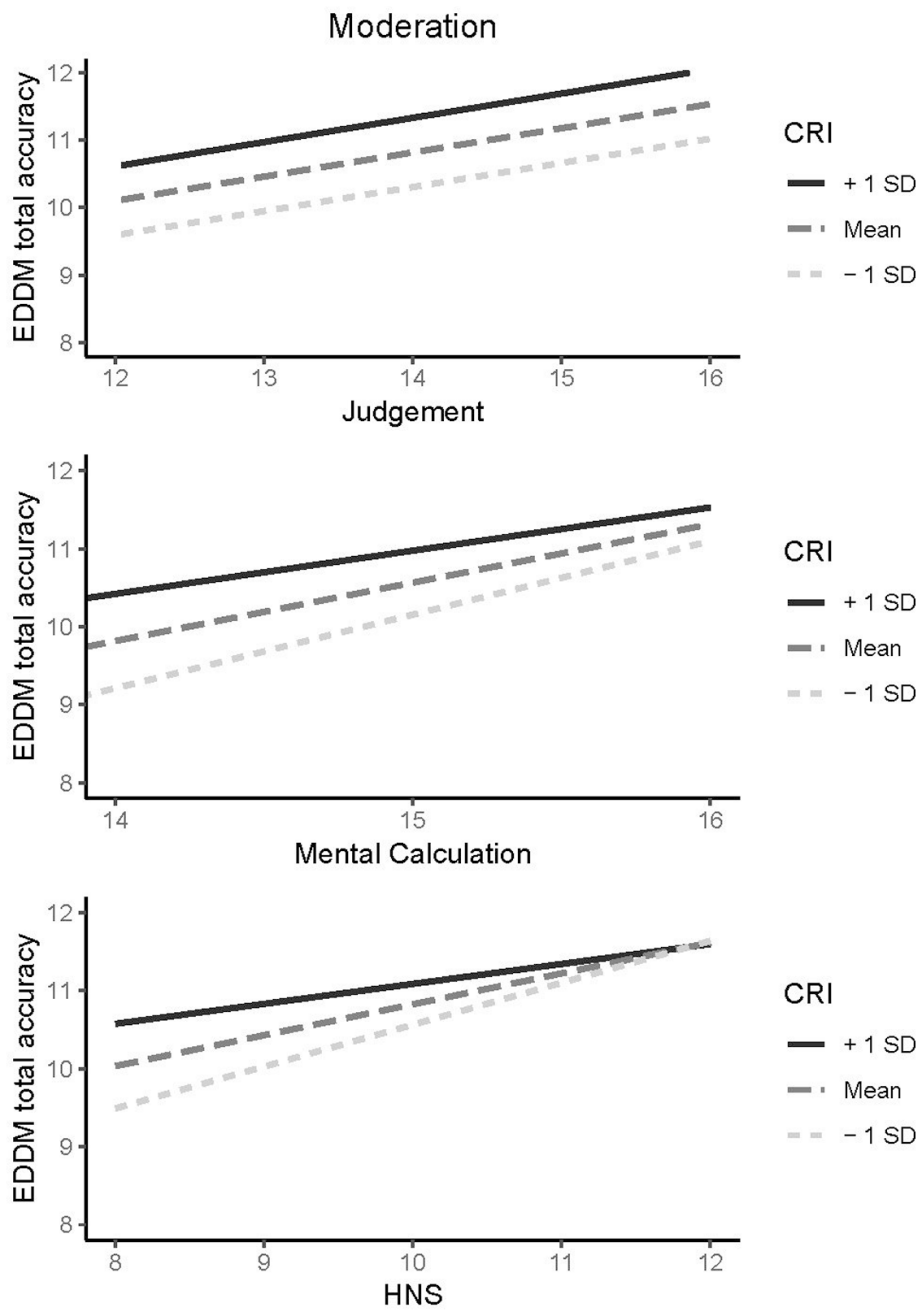
Interactive effects of cognitive reserve, as measured by the CRI, on the relationships between performance on the EDDM and performance on different neurocognitive measures. EDDM, Everyday decision-making task; CRI, Cognitive reserve index; HNS, Health numeracy scale. Dark solid line: high CRI level (+1 SD); dark dotted line: medium-high CRI level (mean); light dotted line: medium CRI level (−1 SD).

In a third moderation analysis, we investigated whether CRI moderates the relationship between health numeracy and decision-making. The overall model was significant [*R^2^* = 0.375, *F*(3, 116) = 27.90, *p* < 0.001]. Health numeracy had a significant main effect, but CRI did not. The interaction was significant, contributing to the explained variance [∆*R^2^* = 0.025, ∆*F*(1,116) = 3.96, *p* = 0.049]. The Johnson-Neyman technique revealed that the effect of health numeracy on decision-making performance was significant when the CRI score was equal to 139.4 or smaller. Health numeracy had a positive effect on decision-making performance at medium (−1 SD; B = 0.54, *t* = 6.20, *p* < 0.001), medium-high (*B* = 0.40, *t* = 6.33, *p* < 0.001), and high CRI levels (+1 SD; B = 0.26, *t* = 2.52, *p* < 0.05; Figure 2).

## 4 Discussion

This study examined the relationships between decision-making, CR, and cognitive functions in midlife and older adults, particularly in the context of processing complex numerical information in everyday decision-making scenarios. Since the decision-making task was specifically designed to investigate congruency and number format effects, we will first summarise performance before discussing CR effects and other results. Participants showed high overall accuracy and a significant congruency effect. Healthy adults aged fifty and older were better at choosing the advantageous option when relevant and irrelevant numerical information aligned (congruent condition) than when they did not (incongruent condition). In congruent trials, both numerical values favoured the advantageous option, making it easier for participants to make the correct choice. Conversely, incongruent trials required more cognitive effort to filter out irrelevant information and focus on the relevant information. As a result, incongruent trials were more challenging and were responded to less accurately. This finding aligns with previous research indicating that tasks presenting conflicting or incongruent information result in higher cognitive load and greater difficulties, as evidenced by slower reaction times and more frequent errors (MacLeod, 2023).

This study also found that overall decision-making performance and the congruency effect were influenced by CR. Higher overall accuracy was associated with higher CR, measured by the CRI (Nucci et al., 2012), which encompasses lifetime curricular and extracurricular education, work experiences, and engagement in intellectually stimulating leisure activities. Notably, individuals with lower CR exhibited a larger congruency effect, responding less accurately to incongruent trials compared to individuals with higher CR, suggesting less efficiency in filtering relevant from irrelevant information. The positive effects of CR on cognitive functioning in older age are well documented, with various measures proposed as proxies (Stern et al., 2020). Our findings suggest that decision-making performance is associated with a comprehensive CR index rather than just years of formal education, aligning with Wilson et al. (2023), who showed that educational attainment alone does not fully explain age-related changes in decision-making. Together, these results emphasize the importance of a broader range of intellectually demanding experiences throughout life and the need to consider a more comprehensive concept of CR—beyond just formal education – when investigating age-related cognitive changes in decision-making.

Overall, decision-making performance was associated with memory, attention, executive functions, numerical competence, and framing effects. CR and measures of judgement, mental calculation, and health numeracy emerged as significant predictors, explaining about 41% of the variance. In a moderation analysis, we found a significant main effect of health numeracy on decision-making performance, as well as a significant interaction between health numeracy and CR. Specifically, higher health numeracy was associated with better decision-making performance across all CR levels, with a stronger association observed among individuals with lower CR. Overall, our findings suggest that individuals with higher CR may show relatively good decision-making performance in everyday scenarios involving complex numerical information, even when their health numeracy skills are low. Consequently, it is reasonable to postulate that midlife and older individuals with relatively lower CR and poorer numeracy skills are at the greatest disadvantage in these contexts. While CR moderated the relationship between health numeracy and decision-making performance, no significant moderation effects were observed for the relationships involving judgement or mental calculation.

Previous research has shown that low numeracy is linked to poorer understanding of treatment risks and benefits (Garcia-Retamero et al., 2019). While highly numerate individuals are more confident with numerical data, those with low numeracy often rely on alternative information sources (Lipkus and Peters, 2009; Peters et al., 2006). Low numeracy also correlates with poorer self-management of chronic diseases (Cavanaugh et al., 2008) and may contribute to higher mortality rates (Reyna et al., 2009). More recent research suggests that low numerate individuals may be more susceptible to misinformation regarding COVID-19 (Roozenbeek et al., 2020). In addition, low numeracy has been linked to poorer financial decision-making (Bangma et al., 2017; Danesin et al., 2022). In general, poorer decision-making performance is associated with lower numeracy (Garcia-Retamero et al., 2019). Our results contribute to this research line by highlighting the potential compensatory role of CR. As suggested by Stern et al. (2020), individuals with greater CR may be more adept at managing challenging tasks and adjust more efficiently as task demands increase. People with low numeracy but relatively higher CR levels may rely on accumulated knowledge, strategies, and experiences gained through education, professional experiences, and intellectually stimulating leisure activities when making informed decisions involving complex numerical information.

Ratio concepts such as frequencies, percentages, fractions, and decimals can be quite challenging not only for children (Thompson et al., 2023), but also for older individuals (Delazer et al., 2013), patients with cognitive impairments (Pertl et al., 2017), and sometimes even highly educated individuals (Lipkus et al., 2001) and healthcare professionals (Gigerenzer et al., 2007). Although focused on a midlife to older sample, we found that overall performance and the number format effect were influenced (albeit marginally) by age. Older age was associated with lower decision-making performance, aligning with Pertl et al. (2017). Accuracy declined for both number formats with age, but the decline was steeper for frequencies than for percentages. Our findings suggest that processing frequencies may become more difficult with age than processing percentages. Pertl et al. (2017) also observed that decision-making performance on percentage items remained relatively stable across age groups, while performance on frequency items was worse among older adults. As Reyna and Brainerd (2008) note, frequencies and percentages do not hold the same psychological significance, even when objectively representing equivalent risk levels. The reasons for differences in the processing of frequencies versus percentages are inconclusive, but factors like individual tendencies toward gist versus verbatim numerical representations, numeracy skills, need for cognition, and interference inhibition may play a role (Reyna and Brainerd, 2008). Consistent with this, our study shows that cognitive aging affects frequency processing more than percentage processing.

In addition to the main findings, two minor results are worth mentioning. First, participants rated their preference for the chosen alternative over the other alternative on a scale from “not at all” to “completely.” An analysis showed a significant positive correlation between decision-making accuracy and preference ratings, aligning with previous research on metacognition and choice confidence (Lee et al., 2023). Choice confidence reflects the subjective belief that the selected option is correct. In healthy individuals, confidence levels often correlate with objective cognitive performance (Lee et al., 2023), although this relationship may vary with age (Overhoff et al., 2022) and across cognitive domains (Meunier-Duperray et al., 2025). Our study found no evidence that this association was age-dependent (analysis not shown). Since our sample included individuals in their fifties and older, excluding younger individuals may have limited our ability to detect age-related changes in this association.

A second minor result pertained to gender. Although males in this study performed slightly more accurately than females, this difference was small and non-significant, offering no reliable support for a gender effect. The existing evidence regarding the influence of gender on decision-making and number processing is inconsistent; some studies have reported differences (Blais and Weber, 2001; Delazer et al., 2013; Villanueva-Moya and Expósito, 2021), while others have not (Schiebener and Brand, 2015; Zamarian et al., 2021). Notably, the observed association between decision-making performance and gender in this study was only marginally non-significant (*p* = 0.056), and thus should be interpreted cautiously. Further studies using the EDDM task are needed before any claims about gender effects can be substantiated.

Some limitations should be considered. First, the cross-sectional design prevents conclusions about how the relationship among decision-making, health numeracy, and CR evolve over time. Future longitudinal studies could provide deeper insights into how decision-making competence and health numeracy changes with age and increasing levels of CR. Second, our sample demonstrated relatively high CRI, which may limit the generalisability of the findings to populations with lower levels of CR. Although the CRI is commonly used as a proxy measure of CR and captures a broader range of life experiences than education alone, it may also partly reflect socioeconomic advantange and greater lifelong opportunities, including access to education, occupational complexity, healthcare availability, and generally healthier living conditions. These factors are closely intertwined with opportunities for cognitive stimulation and may additionally contribute to resilience in aging. Furthermore, this study did not consider other potentially important aspects of CR, such as social engagement or emotional resilience, which may play a particularly relevant role in everyday decision-making among midlife and older adults. Subjective factors, including motivation, which may also influence decision-making performance in later life, were also not considered. Participants in our study performed highly on the decision-making task, with nearly half achieving ceiling-level performance. This was not entirely unexpected, given that the sample consisted of healthy, cognitively high-functioning adults with relatively high CR. Moreover, the EDDM task was designed as a brief, ecologically valid everyday decision-making measure consisting of only 12 items, which may have been relatively easy for individuals with high educational attainment and preserved cognitive functioning. Consequently, the task may have had decreased sensitivity for detecting subtle individual differences among high-performing participants. Furthermore, exact reaction times were not recorded in this study. Future research using the EDDM task may benefit from recording reaction times, as this measure may capture individual differences in cognitive processing efficiency and help differentiate participants who otherwise demonstrate ceiling-level accuracy. Finally, the EDDM task is a novel measure and requires further validation. The fact that this study exclusively involved healthy, cognitively high-functioning adults also limits the generalizability of our findings to clinical populations. Future studies involving samples with greater variability in cognitive functioning and CR, as well as individuals with chronic illnesses or cognitive impairments could provide additional insights into the task’s sensitivity, validity, and broader applicability.

## 5 Conclusion

This study shows that midlife and older adults’ decision-making performance in everyday scenarios involving complex numerical information is associated with cognitive functions such as judgement, mental calculation, and health numeracy, as well as CR. Higher CR – measured in this study by a composite index reflecting curricular and extracurricular education, work experiences, and participation in cognitively stimulating leisure activities – is associated with better decision-making performance in advanced age, particularly in situations presenting incongruent or conflicting information. Higher CR is also associated with reduced negative effects of lower health numeracy skills. Given that older adults are often confronted with life-changing, important decisions, such as choosing the best treatment for a disease, higher CR and stronger cognitive abilities may represent a meaningful advantage. In conclusion, higher levels of CR are linked to reduced age-related declines in health numeracy and lower susceptibility to conflicting, incongruent information during decision-making.

## Data Availability

The raw data supporting the conclusions of this article will be made available by the authors, without undue reservation.
