# Supplementary material for: Everyday decision-making in later life: the role of cognitive reserve and cognitive functions
Source: Front Psychol. 2026 Jun 11;17:1841011. doi: 10.3389/fpsyg.2026.1841011 (PMC13293818; doi:10.3389/fpsyg.2026.1841011)
Supplement: Supplementary file 1 [file Data_Sheet_1.PDF]

Supplementary Materials

1 Additional information about the Everyday Decision-Making Task (EDDM)

The Everyday Decision-Making Task (EDDM) was developed by our working group as a modified version of the decision-making task by Pertl et al. (2017). The EDDM task consists of short text problems in German focusing on practical health decisions (e.g., diets, rehabilitation clinics, sports training programmes). Compared with Pertl et al.'s version, the EDDM task includes a comparable number of congruent and incongruent trials (for examples, see below), yielding a balanced mix of easy and difficult conditions. In addition, the EDDM task focuses on frequencies and percentages, and all numbers are integers (no decimals or fractions are used). The numbers range from 24 to 96, ensuring no difference between conditions with respect to this aspect. Each trial contains two numerical pieces of information: one that is more relevant to the decision (e.g., "Medication A improves symptoms in 95% of cases") and another that is less relevant (hereafter, irrelevant; e.g., "80% of the patients find the name of medication A easy to remember"). Examples of relevant information are the number of patients who reported that the diet was effective, and the number of patients who reported an improvement in quality of life after a stay at a rehabilitation clinic. Examples of irrelevant information are the number of patients describing the neighbourhood of a rehabilitation clinic as nice, and the number of customers who appreciated receiving a Christmas card. The relevant numerical information is presented either as percentages (e.g., 95%) or as frequencies (e.g., 95 out of 100), while the irrelevant information is consistently presented as percentages. In half of the trials, the relevant information is displayed in the upper part of the text for both options; in the other half, it is displayed in the lower part. The relevant and irrelevant information was counterbalanced across option A and B. Based on the results of a pilot study with 32 items distributed evenly across the four conditions (congruent percentage, congruent frequency, incongruent percentage, incongruent frequency), we selected 12 items (three per condition) for which participant accuracy was neither too high nor too low. This selection aimed to avoid items that were too difficult or too easy. The difference in accuracy between percentage- and frequency-based items was 8.2% in the congruent condition and 8.8% in the incongruent condition. In this study, the order of trial presentation was fixed across participants: no more than three consecutive trials displayed the relevant information in the same position (above or below), and no more than two consecutive trials belonged to the same congruency condition. PowerPoint was used to present the EDDM task.

For validation purposes, the 12 items were presented to 10 independent raters, who assessed the relevance of each piece of information for the decision. The item-level CVI ranged between 0.90 and 1, while the scale-level CVI was 0.98, indicating a high level of agreement across items. In a further sample of 158 participants (aged 20–90 years), the EDDM task demonstrated acceptable internal consistency (Cronbach's alpha = 0.723).

1.1 Examples for congruent and incongruent items in the Everyday Decision-Making Task (EDDM)

Congruent item:

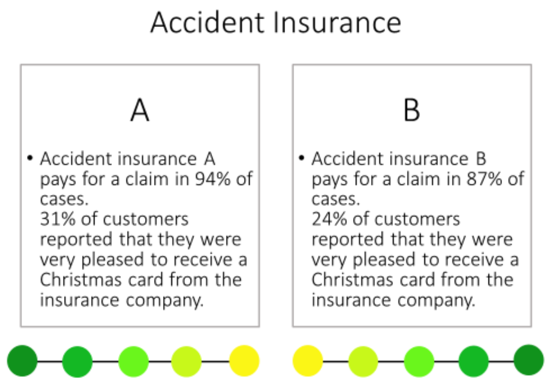

Incongruent item:

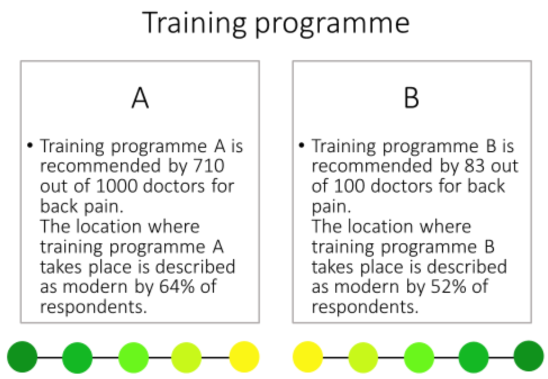

2 Description of screening tests and questionnaires

Participants completed screening tests assessing their global cognitive status (Mini Mental State Examination, MMSE; Berres et al., 2000; Folstein et al., 1975) and reading comprehension (Delazer et al., 2013). They also completed the German adaptation of the Hospital Anxiety and Depression Scale

(HADS-D; Zigmond and Snaith, 1983) to identify pronounced depressive and anxious symptoms, which could influence performance on cognitive tests.

3 Description of neuropsychological background tests and numeracy tasks

Participants underwent a comprehensive neuropsychological assessment battery to exclude potential cases of pathological cognitive decline and to examine possible associations with decision-making. Their performance was compared to published norms; none of the participants exhibited evidence of such decline. Specifically, participants completed the CERAD-plus test battery (Consortium to Establish a Registry for Alzheimer's Disease; Berres et al., 2000), which includes tests assessing global cognitive status (MMSE), verbal memory (learning, free recall, recognition), figural memory (free recall), visuo-construction (copying of figures), naming to confrontation (Boston Naming Test short), semantic and phonemic verbal fluency (animals/min and s-words/min), psychomotor speed (Trail Making Test part A, TMT-A) and cognitive flexibility (TMT-B). Additionally, participants completed tests for planning and conceptualization (CLOX 1; Royall et al., 1998), verbal attention span (digit span forward, WAIS-IV; Petermann, 2012), verbal working memory (digit span backward, WAIS-IV; Petermann, 2012), interference inhibition (Farb-Wort-Test, NAI; Oswald and Fleischmann, 1999), and everyday knowledge and judgement (subtest judgement, NAB; Petermann et al., 2016). Moreover, participants completed a cognitive reflection test (CRT; Frederick, 2005), a framing task (Zamarian et al., 2010), a mental calculation task (Delazer et al., 2013) and a health numeracy task (Lipkus et al., 2001; adapted by Delazer et al., 2013). In the CRT (Frederick, 2005), participants are presented with three numerical text problems, requiring them to suppress intuitive responses and engage in more deliberative thinking. The framing task (Zamarian et al., 2010) consists of 20 statements related to outcomes of fictitious medications for mild diseases, which have to be evaluated on a 7-point coloured scale from negative (left side, red) to positive (right side, green). In half of the items, the statement is presented in positive terms (e.g., successful in 84% of cases), while the other half is phrased in negative terms (e.g., no improvement in 16% of cases). The framing effect is determined by comparing scores between positively and negatively framed items. In this study, performance was measured by the difference in these scores, with a value above zero indicating that participants rated positively phrased items more favourable than negatively phrased items. Mental calculation was assessed with 16 arithmetic problems (4 additions, 4 subtractions, 4 multiplications and 4 divisions) with increasing difficulty (Delazer et al., 2013). The Health Numeracy Scale (HNS) is an adapted version from Lipkus et al. (2001) (for details see Delazer et al., 2013). It consists of 12 items relating to health and risk

contexts, where participants have to convert proportions or percentages, evaluate risks, perform arithmetic operations, and determine the appropriate dosage of a medication from a short patient leaflet.

4 Results in the screening assessment and in the comprehensive neuropsychological background assessment

Results are reported in Table S1. In the MMSE, all participants had a global score of at least 27. In the reading comprehension screening test, all but four participants performed at ceiling. In a questionnaire on current anxiety and depression symptoms, two participants obtained scores higher than 10 on the anxiety scale. None of these participants reported a current or past psychiatric diagnosis. Therefore, all participants were included in the sample. In all cognitive tests, group scores were in the average range of standardised norms. At the single-subject level, none of the participants exhibited evidence of pathological cognitive decline.

Table S1: Scores in the screening evaluation and in the comprehensive neuropsychological background assessment.

	Max. score	M	SD	Min.	Max.
**Screening tests**					
MMSE[a]	30	29.4	0.8	27	30
Reading comprehension	6	6.0	0.2	5	6
Anxiety	21	4.5	2.7	0	12
Depression	21	3.0	2.4	0	10
**Neuropsychological background tests**					
Verbal Attention Span	16	9.8	2.2	6	16
Verbal Working Memory	16	8.9	2.1	4	16
Verbal Learning[a]	30	21.8	3.7	13	29
Verbal Free Recall[a]	10	7.8	1.7	2	10
Figural Free Recall[a]	14	10.6	2.1	3	14
Copying of Figures[a]	11	10.6	0.9	6	11
Object Naming to Confrontation[a]	15	14.9	0.4	13	15
Semantic Verbal Fluency[a] (animals/min)		25.3	5.9	15	43
Phonemic Verbal Fluency[a](s-words/min)		14.9	3.9	6	26
Psychomotor Speed (TMT A[a]) (s)		35.7	14.1	14	84
Cognitive Flexibility (TMT B[a]) (s)		78.8	36.8	35	226
Planning/Conceptualisation (CLOX1)	15	13.2	2.0	1	15
Interference Inhibition (s)		18.4	9.8	4	65
Judgement	16	14.1	1.5	9	16
Cognitive Reflection Test	3	1.0	1.1	0	3
Framing		1.1	1.3	-2	6
Mental Calculation	16	15.4	0.9	13	16
Health Numeracy Scale	12	10.3	1.8	3	12

Notes. Max. score = maximum possible score; M = mean; SD = standard deviation; Min. = minimum; Max. = maximum; MMSE = Mini Mental State Examination; TMT = Trail Making Test; min = minutes; s = seconds. [a]Test from the CERAD battery.

Table S2: Accuracy scores and median preference scores in the EDDM.

	Max. score	M	SD	Min.	Max.
Total accuracy score	12	10.9	1.5	5	12
Accuracy in congruent trials	6	5.8	0.6	3	6
Accuracy in C F	3	2.9	0.4	1	3
Accuracy in C P	3	2.9	0.3	1	3
Accuracy in incongruent trials	6	5.1	1.2	0	6
Accuracy in IC F	3	2.6	0.7	0	3
Accuracy in IC P	3	2.5	0.7	0	3
Total preference score	5	4.4	0.7	1	5
Preference score in congruent trials	5	4.4	0.7	1	5
Preference score in C F	5	4.4	0.8	1	5
Preference score in C P	5	4.6	0.8	1	5
Preference score in incongruent trials	5	4.2	0.8	1	5
Preference score in IC F	5	4.2	0.8	1	5
Preference score in IC P	5	4.1	0.9	1	5

Notes. EDDM = Every Day Decision-Making Task; Max. score = maximum possible score; M = mean; SD = standard deviation; Min. = minimum; Max. = maximum; C = congruent; IC = incongruent; F = frequency; P = percentage.

Figure F1: Correlations between total accuracy scores in the EDDM and neurocognitive variables.

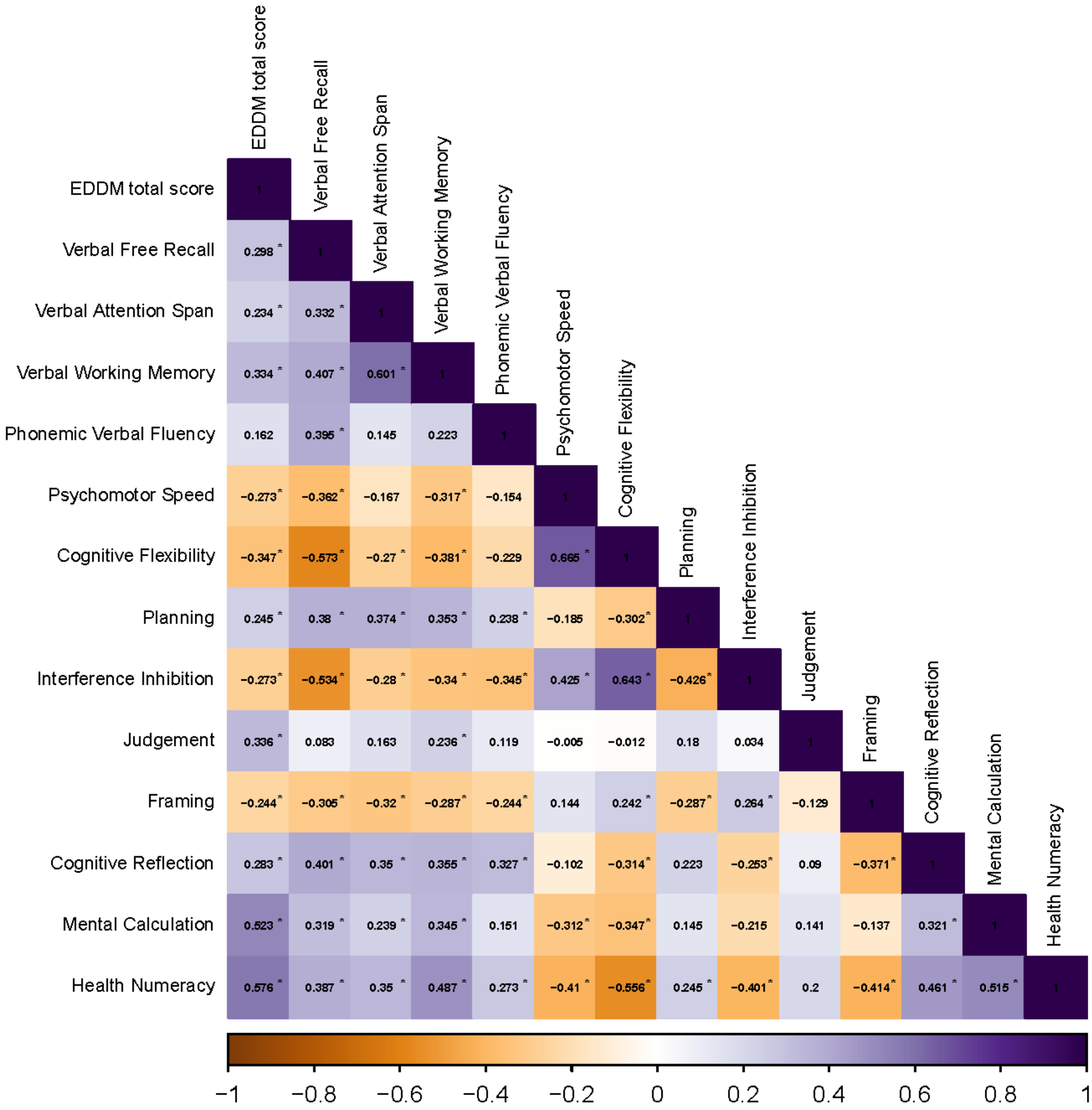

Notes. EDDM = Every Day Decision-Making Task.

*p<.05 after FDR correction.

5 References

Berres, M., Monsch, A. U., Bernasconi, F., Thalmann, B., and Stähelin, H. B. (2000). Normal ranges of neuropsychological tests for the diagnosis of Alzheimer's disease. *Studies in health technology and informatics* 77, 195–199.

Delazer, M., Kemmler, G., and Benke, T. (2013). Health numeracy and cognitive decline in advanced age. *Neuropsychology, development, and cognition. Section B, Aging, neuropsychology and cognition* 20, 639–659. doi: 10.1080/13825585.2012.750261.

Folstein, M. F., Folstein, S. E., and McHugh, P. R. (1975). "Mini-mental state". A practical method for grading the cognitive state of patients for the clinician. *Journal of psychiatric research* 12, 189–198. doi: 10.1016/0022-3956(75)90026-6.

Frederick, S. (2005). Cognitive reflection and decision making. *Journal of Economic Perspectives* 19, 25–42. doi: 10.1257/089533005775196732.

Lipkus, I. M., Samsa, G., and Rimer, B. K. (2001). General performance on a numeracy scale among highly educated samples. *Medical decision making: an international journal of the Society for Medical Decision Making* 21, 37–44. doi: 10.1177/0272989X0102100105.

Oswald, W. D., and Fleischmann, U. M. (1999). *Nürnberger-Alters-Inventar.* 4. edition. Goettingen: Hogrefe.

Pertl, M.-T., Benke, T., Zamarian, L., and Delazer, M. (2017). Effects of healthy aging and mild cognitive impairment on a real-life decision-making task. *Journal of Alzheimer's disease : JAD* 58, 1077–1087. doi: 10.3233/JAD-170119.

Petermann, F., ed. (2012). *WAIS-IV: Wechsler adult intelligence scale - fourth edition: German adaptation after David Wechsler.* Frankfurt/M. Pearson Assessment & Information.

Petermann, F., Jäncke, L., and Waldmann, H.-C. (2016). *Neuropsychological Assessment Battery: German adaptation of the Neuropsychological Assessment Battery (NAB) after Robert A. Stern and Travis White.* Goettingen: Hogrefe.

Royall, D. R., Cordes, J. A., and Polk, M. (1998). CLOX: An executive clock drawing task. *Journal of neurology, neurosurgery, and psychiatry* 64, 588–594. doi: 10.1136/jnnp.64.5.588.

Zamarian, L., Benke, T., Buchler, M., Wenter, J., and Delazer, M. (2010). Information about medications may cause misunderstanding in older adults with cognitive impairment. *Journal of the neurological sciences* 298, 46–51. doi: 10.1016/j.jns.2010.08.061.

Zigmond, A. S., and Snaith, R. P. (1983). The hospital anxiety and depression scale. *Acta psychiatrica Scandinavica* 67, 361–370. doi: 10.1111/j.1600-0447.1983.tb09716.x.
